# Disentangling psychopathology, substance use and dependence: a factor analysis

**DOI:** 10.1186/s12888-016-0988-1

**Published:** 2016-08-08

**Authors:** Jaime Delgadillo, Jan R. Böhnke, Elizabeth Hughes, Simon Gilbody

**Affiliations:** 1 Leeds Community Healthcare NHS Trust, Leeds, UK; 2 Hull York Medical School and Department of Health Sciences, University of York, York, UK; 3 University of Huddersfield and South West Yorkshire Partnership NHS Foundation Trust, Huddersfield, UK

**Keywords:** Depression, Anxiety, Alcohol, Drugs, Addiction

## Abstract

**Background:**

The notion that substance use can induce symptoms of depression and anxiety is influential in clinical practice, however questions remain about the empirical support for this hypothesis.

**Methods:**

We analysed mental health and substance dependence screening records for 280 outpatients in addictions treatment. Item-level data for depression (PHQ-9), anxiety (GAD-7), severity of dependence (SDS) and self-reported weekly substance use were studied using factor analysis and correlations. Symptom-level associations between substance use and psychological distress symptoms were examined after controlling for underlying levels of psychopathology.

**Results:**

We obtained a two-factor solution accounting for approximately 48 % of total variance. Depression and anxiety symptoms loaded onto a single psychopathology factor. Severity of dependence (SDS) and substance use measures loaded onto a distinct but correlated factor. After controlling for latent levels of psychopathology, the only remaining symptom-level associations were impaired concentration linked to cannabis use and irritability linked to alcohol use. Dependence (SDS) was prominently associated with depressive rumination, and negatively correlated with residual anxiety symptoms related to substance use (e.g., craving).

**Conclusions:**

Overall, this analysis supports a psychological understanding of comorbidity; with dependence, craving, negative reinforcement and rumination as key variables.

## Background

Substance use disorders and common mental disorders (CMD) such as depression and anxiety often co-exist. This finding is consistent across epidemiological surveys conducted in the general population [1–4] and in clinical samples [5–7]. Many have interpreted this common overlap in disorders as evidence that substance use can induce or mimic depressive and anxiety symptoms. The notion of substance induced disorders has a firmly anchored place in diagnostic manuals [8, 9], structured assessment interviews [10–12] and clinical practice guidelines in the addictions field [13–15]. An illustrative example of the reification of this assumption is offered by Raimo and Schuckit who assert that –when assessing alcohol users– “unless independent major depressive syndromes are clearly established, it is assumed that the only depressive episodes that have been experienced were likely to be substance induced” [16], p. 935.

Although the substance induced hypothesis continues to influence current clinical practice, there is some contention about its empirical foundations. More recent epidemiological surveys [17, 18] found that substance induced depression and anxiety disorders are less common than expected (≤10 % of cases in clinical samples, and <1 % in the general population). Several clinical reports have noted that substance users’ psychological distress symptoms remitted after a brief period of observation, typically within less than a month [19–21]. Such reports are taken to indicate that a reduction of psychoactive substance use may account for improved psychological state, but they do not account for or rule out the possible influence of other ‘third’ or potentially mediating variables. For example, large cross-sectional and longitudinal surveys have demonstrated that this combination of disorders appears to be mostly prominent in people who meet criteria for alcohol or substance dependence, rather than in those with less chronic or episodic substance use [22, 23]. This raises questions about whether there is indeed a causal relationship between levels of substance use and symptoms of CMD. An alternative possibility is that substance dependence as a psychological and behavioural syndrome may be associated with CMD, irrespective of the level of consumption. Severity of dependence is known to correlate with level of substance use and psychopathology; however symptoms of dependence can nevertheless be present in abstainers [24].

Furthermore, most studies in this area examine associations at the level of syndromes or disorders, which could possibly mask specific symptom-level relationships and could also introduce artificial relationships because of double counting of symptoms [25]. For instance, in a recent study, severity of dependence has been found to correlate with several symptoms of depression and anxiety, whereas only few CMD symptoms were correlated with level of substance use and abstainers were just as likely to have a CMD diagnosis compared to users [26]. Symptom-level associations such as these have rarely been examined, and until such studies proliferate the debate about causal links will probably continue unabated.

In the present paper, we examine the relationships between substance use, severity of dependence and CMD at the level of symptoms and latent dimensions of psychopathology. The main aims of this study were to determine the factor structure of a battery of measures covering the above domains in a clinical sample, and to describe and interpret the patterns of symptom-level associations.

## Methods

### Design and context

This paper presents factor analyses and correlations based on pooled data from two prior studies that applied a mental health screening strategy in addiction services [27, 28]. Both studies recruited patients accessing community based alcohol, drug and rehabilitation teams in a large city in the north of England. These were multi-disciplinary teams offering access to medical (e.g., opiate substitute treatment) and psychosocial input to minimise harms associated with substance use and to support access to housing, social care, training, peer mentorship and employment opportunities. Interventions offered by these services were consistent with national guidelines for the management of substance use disorders [29, 30].

Both studies purposefully aimed to screen patients at various stages of their contact with addiction services (range of months in treatment = 0–70), which would ensure results were less likely to be confounded by the acute distress typically observed in new patients. Both studies enlisted the support of drug and alcohol workers to screen participants, and applied methods to minimise selection bias (database searches, use of appointment and reminder systems to prompt clinicians to contact potential participants). Both studies applied the same patient reported outcome measures (PROMs) to identify patients with CMD. Further details of the primary studies and recruitment methods can be found in the source publications.

Informed consent was provided by all participants across both studies to use their anonymous data as part of research, and ethical approval was provided by the University of York [reference: RGC/03.07.09] and the English National Health Service Research Ethics Committee [REC reference: 12/YH/0096].

### Psychometric measures and screening strategy

Four PROMs were applied to screen for substance use, dependence and symptoms of common mental health problems.

#### Addiction related measures

The Treatment Outcomes Profile (TOP) is a twenty-item measure covering four domains: substance use, injecting risk behaviour, crime and health & social functioning [31]. The measure is based on the timeline follow-back method [32]; which prompts respondents to recall the average quantity and frequency of substances used during the last 4 weeks. The TOP has been reported to have adequate sensitivity (0.57–0.89) and specificity (0.85–0.92) compared to independent drug toxicology tests.

The Severity of Dependence Scale (SDS) is a measure of psychological dependence, capturing aspects of compulsive substance use, concern over use and degree of control over use [33]. This five-item questionnaire yields a total score between 0 and 15, where scores above 10 are indicative of severe dependence. The SDS has been extensively validated as a reliable screening tool for dependence on a variety of substances including alcohol, heroin, crack, cannabis and other illicit and prescription drugs [34–38]. Patients in the source studies were asked to complete the SDS measure only for their primary substance of concern (most frequently used and/or the index substance for which they sought treatment in the case of abstainers). The internal reliability (Cronbach’s alpha) estimate for the SDS in the study sample was α = .85.

#### Common mental disorder (CMD) related measures

The PHQ-9 is a nine-item questionnaire based on diagnostic criteria for major depression, which renders a severity score between 0 and 27 [39]. The PHQ-9 has been validated as a reliable case-finding tool for clinically significant depression symptoms in substance users based on a cut-off score ≥12, with 81 % sensitivity and 75 % specificity [27]. Cronbach’s alpha for PHQ-9 in this study sample was α = .83.

The GAD-7 questionnaire [40] was used to assess severity of anxiety symptoms, with scores ranging between 0 and 21, where a score ≥9 indicates the likely presence of an anxiety disorder with 80 % sensitivity and 86 % specificity [41]. Cronbach’s alpha for GAD-7 in this study sample was α = .88.

The TOP questionnaire cited above also contains a single item psychological health scale (TOP-4a), which asks respondents to rate their psychological wellbeing on a scale of 0 (poor) to 20 (good). A cut-off score ≤12 on the TOP-4a has been found to have adequate sensitivity (83 %) and specificity (71 %) to detect a probable diagnosis of a CMD [42].

### Sample characteristics

A total of 280 screening records were included in this study. Most respondents were white British (80.3 %) males (74.5 %), with a mean age of 36.74 (SD = 7.18; range = 23 – 60). The majority (97.1 %) were prescribed opiate substitute medication, but less than half (40.7 %) were prescribed antidepressants. The most commonly used substances in this sample were alcohol (47.1 % of users), heroin (42.9 %), cannabis (23.9 %), crack (23.2 %), benzodiazepines (6.4 %), cocaine (3.6 %), and amphetamines (2.1 %). Approximately 47.5 % were polysubstance users, 17.5 % reported intravenous use, and 17.5 % reported being currently abstinent for at least a month. Weekly substance use estimates are presented in Table 1 for the four most commonly used substances (alcohol, heroin, cannabis and crack). Mean scores for PROMs were SDS = 7.08 (SD = 4.69); PHQ-9 = 13.58 (SD = 6.14); GAD-7 = 10.28 (SD = 5.58); TOP-4a = 9.95 (SD = 4.29). According to mental health measures, 61.8 % had clinically significant depression (PHQ-9) symptoms, 59.2 % had clinically significant anxiety (GAD-7) symptoms, and 73.5 % were likely to meet criteria for a CMD (TOP-4a).

**Table 1:** Self-reported substance use variables derived from Treatment Outcomes Profile (TOP)

	QF measures			Distribution of cases across quintile levels of substance use				
Substances	Mean	SD	Range	Non-user	Light	Moderate	Frequent	Heavy
Alcohol (u)	40.43	53.72	1 – 252	136	32	19	21	24
Heroin (g)	0.91	1.51	0.01 – 10.50	148	35	10	22	17
Cannabis (j)	16.65	16.25	1 – 70	188	11	13	13	7
Crack (g)	0.47	0.68	0.02 – 3.50	191	13	9	10	9

*Notes*: *QF* quantity x frequency per week; alcohol measured in units (u); heroin and crack measured in grams (g); cannabis measured in joints (j)

### Statistical analyses

Consistent with the aims of this study, statistical analyses were performed in 3 stages focusing on (1) describing the sample, (2) undertaking factor analyses, and (3) exploring associations between screening domains. The following analyses were performed with IBM SPSS 22 and FACTOR 9.3.1.

Descriptive statistics are reported for demographic characteristics, and mean levels of substance use, severity of dependence, depression and anxiety symptoms. To enhance the precision of substance use information gathered by the TOP questionnaire, we calculated quantity x frequency (QF) measures by multiplying the average amount of use in a typical day by the number of days used in the last week. This method is likely to reduce recall bias and has been used in prior correlational studies [43]. Given the sample size limits, QF measures were only derived for the 4 most commonly used substances: alcohol, heroin, crack, cannabis.

Two datasets including all items (*N* = 26) across PHQ-9, GAD-7, SDS, TOP-4a and QF measures were used for factor analyses. Dataset A included 232 (82.9 %) cases with complete data on all items. Dataset B included all cases (*N* = 280), where missing items were imputed using an expectation-maximization procedure [44].

Conventional analyses were used to empirically evaluate the adequacy of the dataset for factor analysis; these included the Kaiser-Meyer-Olkin (KMO) test and Bartlett’s test of sphericity. Since we analysed ordinal questionnaire responses, polychoric correlations were used to determine the degree of relationship between the individual items. In contrast to Pearson correlations, polychoric correlations assume a monotone relationship between two variables and the response categories are not assumed to be equidistant across items [45]. Since the QF data were highly skewed and not of the same magnitude across the four different substances, we transformed them into 5 ordinal variables. The new variables differentiate between zero (abstainers) versus four increasing levels of consumption (4 quartile levels of substance use displayed in Table 1). This transformation is clinically meaningful, the responses are scaled on a similar metric to each other, and therefore it enabled us to analyse these variables together with the items of the other clinical measures [46].

Our *a priori* assumptions were that we would find multiple correlated factors: SDS, a single factor for CMD symptoms, and 3 factors for QF measures (one for alcohol, one for cannabis, and one for heroin and crack given this common polysubstance use pattern). Based on this rationale, we performed factor analysis based on unweighted least squares, with promin rotation [47]. Next, we applied Parallel Analysis (PA) [48] and the Hull method [49] to empirically determine how many factors optimally explained the variability in the data. While parallel analysis determines the number of factors that extract more variability than expected in a structurally similar set of random data, the Hull Method determines a solution balancing complexity (degrees of freedom of a solution) and fit to the data (comparative fit index). This analysis strategy was initially performed in dataset A, and replicated in dataset B as a sensitivity analysis.

Finally, we examined item-level correlations between the domains of substance use and CMD symptoms in dataset A. These were carried out as partial correlations controlling for the CMD latent dimension scores derived from the factor analysis. The rationale for this was to investigate whether any associations between substance use and CMD symptoms remain after controlling for psychopathology. In a second step, these partial correlations additionally controlled for the potential influence of 'recent quitters' who abstained during 4 weeks and may be displaying atypical levels of distress [50]. Instances with discrepant results between steps 1 and 2 of analysis prompted us to investigate possible non-linear associations graphically (using error bars) and statistically (using polynomial analysis of variance equations).

## Results

### Factor analysis

#### Assumption testing

The suitability of applying factor analysis with the set of 26 variables in dataset A was confirmed by Bartlett’s test of sphericity, which was non-significant (approximate *x*^2^ = 2524.8, df = 325, *p* < .001). In addition, the overall KMO measure of sampling adequacy was 0.90, indicating excellent factorability.

#### Factor structure

In contrast to our expectations, both selection criteria suggested a two factor solution: Two inter-correlated (*r* = .45) latent factors accounted for 47.5 % of variance in the dataset (35.1 % and 12.4 %, respectively). After promin rotation, factor 1 included item TOP-4a and all items from PHQ-9 and GAD-7 (loadings = .50 − .93), thus representing a latent CMD factor. TOP-4a was negatively correlated (−.50) with factor 1, since a higher score on this measure indicates better psychological wellbeing. Factor 2 included all SDS items (loadings = .61 − .90), plus QF measures for heroin (.53) and crack use (.36), representing a substance use and dependence (SUD) dimension. QF measures for alcohol (factor 1 loading = .02, factor 2 = .16) and cannabis (factor 1 loading = −.11, factor 2 = .17) were excluded from the final rotated solution, given their weak correlations which were smaller than the conventional cut-off of .30.

Repeating these series of analyses in sample B led to similar results. A two factor solution with the same item loading structure explained 48.2 % of variance (factor 1 = 35.5 %, factor 2 = 12.7 %) and the inter-factor correlation was .43. Therefore, missing data items were unlikely to influence the results of the analysis. The final rotated solutions attained in both samples are displayed in Table 2.

**Table 2:** Factor structure of common mental disorder (CMD), substance use and dependence (SUD) screening tools

		Sample A (*N* = 232)			Sample B (*N* = 280)		
		Inter-factors correlation = .45			Inter-factors correlation = .43		
Item	Description	Factor 1 (CMD)	Factor 2 (SUD)	Communality	Factor 1 (CMD)	Factor 2 (SUD)	Communality
Alcohol QF	Alcohol quantity x frequency of use (last week)	–	–	.03	–	–	.02
Heroin QF	Heroin quantity x frequency of use (last week)		.53	.27		.56	.28
Crack QF	Crack quantity x frequency of use (last week)		.36	.11		.39	.14
Cannabis QF	Cannabis quantity x frequency of use (last week)	–	–	.03	–	–	.02
TOP4a	Self-rated psychological wellbeing	-.50		.25	-.54		.27
PHQ1	Loss of interest and pleasure	.53		.33	.59		.37
PHQ2	Depressed mood	.80		.63	.82		.63
PHQ3	Disrupted sleeping patterns	.54		.28	.58		.31
PHQ4	Lethargy	.58		.34	.57		.31
PHQ5	Disruptions in appetite	.52		.32	.54		.32
PHQ6	Depressive rumination	.57		.58	.61		.60
PHQ7	Disrupted concentration	.61		.33	.67		.39
PHQ8	Psychomotor deficits or agitation	.55		.37	.59		.40
PHQ9	Suicidal ideation	.61		.45	.62		.48
GAD1	Feelings of anxiety and nervousness	.79		.58	.81		.62
GAD2	Impaired control over worry	.93		.74	.88		.70
GAD3	Generalised worry about different things	.83		.62	.82		.62
GAD4	Trouble relaxing	.84		.62	.86		.66
GAD5	Restlessness	.68		.50	.69		.51
GAD6	Irritability	.55		.37	.57		.40
GAD7	Fear	.73		.56	.63		.46
SDS1	Impaired control over substance use		.90	.76		.88	.74
SDS2	Worry about missing a dose of substance use		.78	.58		.76	.55
SDS3	Worry about substance use		.87	.78		.87	.79
SDS4	Wish to stop substance use		.83	.62		.84	.64
SDS5	Difficulty in abstaining from substance use		.61	.41		.62	.41

*Notes*: Factor loadings smaller than cut-off of .30 were omitted. Sample A included all cases with complete data on all items. Sample B applied multivariate imputation of missing data items. Variance explained by the factor solution was 47.5 % in sample A and 48.2 % in Sample B

### Partial correlations controlling for level of (latent) CMD psychopathology

**Fig. 1:**
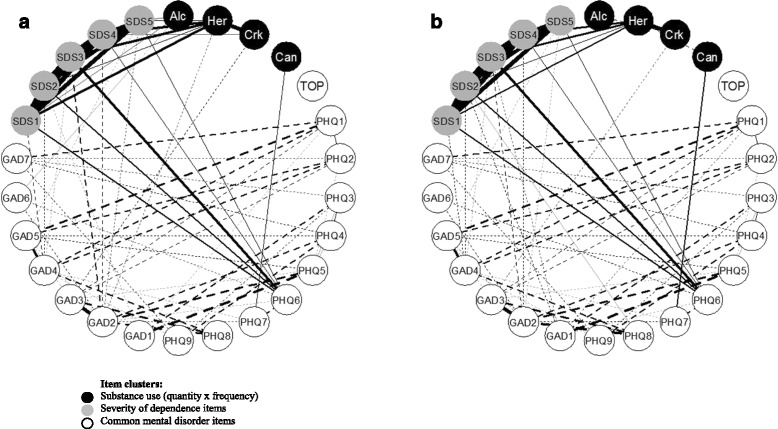
Network plots of item-level partial correlations controlling for latent psychopathology. **a** Controlling for common mental disorders (CMD). **b** Controlling for CMD and abstainers. Notes: Only statistically significant partial correlations shown (*p* <.05); Solid lines = positive correlations, dashed lines = negative correlations; Thickness of lines = strength of correlations; Her = heroin; Crk = crack; Alc = alcohol; Can = cannabis; TOP = Treatment Outcomes Profile item 4a (psychological distress)

Figure 1 displays network plots of item-level partial correlations from the two steps of analysis; where solid and dashed lines represent positive and negative correlations respectively. We observed small residual correlations between PHQ-9 and GAD-7 items after partialling out the shared CMD factor which explained a large proportion of variance in both measures. However, contrary to our expectations, we noted several negative partial correlations between these measures in both steps of analysis (*r* = −.13 to–.28).

All SDS items were significantly correlated with PHQ-9 item 6 (depressive rumination; *r* = −.19 − .31) and negatively correlated (*r* = −.13 to–.22) with GAD7 items 1 (anxiety), 2 (uncontrollable worry), 4 (trouble relaxing), and 5 (restlessness). All SDS items were correlated with heroin use (*r* = .16 − .29), although item 5 (difficulty abstaining) was no longer significant in the second step of analysis after controlling for abstainers.

**Fig. 2:**
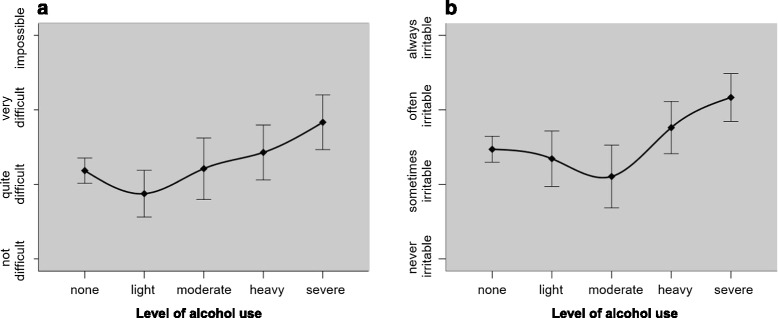
Non-linear associations between alcohol use, dependence and anxiety symptoms. **a** Difficulty in abstaining from alcohol use. **b** Alcohol use and feelings of irritability

Alcohol use was correlated with GAD-7 item 6 (irritability; *r* = .14) although this relationship was not significant after controlling for abstainers. After closer examination, we found that a v-shaped non-linear equation offered an adequate fit to this relationship; F (4, 227) = 4.17, *p* < .01, weighted quadratic term *p* < .01. Heroin use was negatively correlated (*r* = −.15) with GAD-7 item 2 (uncontrollable worry), but this was not significant at step 2. A non-linear equation did not improve model fit for this relationship; F (4, 227) = 0.30, *p* = .88. Crack use was negatively correlated (*r* = −.15 to–.17) with GAD-7 item 3 (generalised worry). Cannabis use was correlated (*r* = .19– .22) with PHQ–9 item 7 (disrupted concentration). As expected, heroin and crack use were significantly correlated (*r* = .37 − .40). After controlling for abstainers, we observed negative correlations between alcohol and crack use (*r* = −.19), and heroin and cannabis use (*r* = −.20). In these inter-substance correlations, the more appropriate analysis was step 2, since logically we are investigating likelihood of poly-use for those who actively use substances. Figure 2 displays significant non-linear associations between alcohol with irritability (GAD-7 tem 6, *r* = −.19) and with difficulty abstaining (SDS item 5, *r* = −.19).

## Discussion

### Main findings

This study presents a detailed examination of symptom-level associations between common mental disorders (CMD), substance use and severity of dependence. To our knowledge, such analyses are rare [26], and offer novel information to validate theories about comorbidity and psychopathological processes. Consistent with the wider literature cited above, we found significant and moderate correlations between psychopathology and severity of dependence in a clinical sample of drug and alcohol users, although these were distinct and separate dimensions. Factor analyses indicated that depression and anxiety symptoms loaded onto a single underlying dimension, which converges with prior studies [51, 52]. Substance use and severity of dependence were strongly associated and mapped onto a single dimension. After controlling for patients’ level of psychopathology represented by their CMD factor scores, we found evidence of statistically significant albeit small correlations between substance use and psychological distress at the level of individual symptoms. In what follows, we describe these patterns of associations and offer a theoretical interpretation of the findings.

The most prominent association pattern was that between severity of dependence (SDS) with depressive rumination, and its negative correlation with feelings of anxiety, uncontrollable worry, restlessness and inability to relax. This inverse pattern may explain the unexpected residual negative correlations between depression and anxiety symptoms (after partialling out their shared psychopathology). We note that these residual anxiety symptoms are similar to the phenomenology of craving and withdrawal [8, 9]. Further supporting evidence was found in the partial correlations (controlling for psychopathology) between worry about missing a dose (of substance use) and psychomotor agitation, impaired control over substance use and inability to relax, and the finding that heavier heroin and crack users were less prone to worry. It is plausible that more severely dependent respondents were compulsively using substances in a way that mitigated such feelings of restlessness and craving, but this ultimately resulted in negative bouts of rumination, which in turn exacerbated wider aspects of depressive mood.

This deduction from our findings fits within a wider body of research. For example, Franken et al. [53] carried out a factor analysis of two opiate craving questionnaires in a sample of 102 addiction service users and described a 3-factor solution covering aspects of ‘desire and intention to use’ , ‘negative reinforcement’ and ‘control’. The strongest inter-factor correlation (*r* = .39) indicated that ‘desire to use’ substances was driven by a need to suppress worry, tension and thoughts about life problems (‘negative reinforcement’). Furthermore, items from the ‘control’ factor were negatively associated with items from the other 2 domains. Craving is also known to be associated with biased attention to environmental cues related to substance use [54], which may partly explain the experience of uncontrollable worry (about substance use). On the other hand, perseverant negative thinking (rumination) is often associated with depression [55–57] and has been described as a transdiagnostic factor underlying several mental disorders [58]. Rumination has been found to predict the onset of depressive symptoms in non-clinical samples [59–61], and it appears to maintain symptoms of low mood and anxiety in depressed patients [62].

Moreover, we found that two of the substance use measures (heroin and alcohol) were correlated with loss of control over substance use. This is consistent with research indicating that alcohol impairs inhibitory control [63, 64], although this is less evident for opiates [64] and instead may reflect a psychological aspect of lower perceived control over heroin use. Cannabis use was correlated with impaired concentration, which reflects the expected psychoactive effects of this substance [65]. Although robust quantitative research for cannabis-induced neurocognitive deficits is still scarce, there is some evidence that cannabis use impairs the ability to learn and recall new information [66].

A particularly interesting pattern of non-linear associations were found for alcohol use (Fig. 2). Moderate drinkers had lower mean scores for feelings of irritability compared to non-drinkers and heavy/severe users. In addition, light drinkers reported lower levels of perceived difficulty in abstaining compared to non-drinkers and moderate to severe drinkers. Non-linear associations between alcohol and psychological distress have been reported in numerous studies [50, 67–72]. These studies model associations based on aggregated scores, which may mask more specific symptom-level patterns. The ‘stress buffer’ theory [73] seems like a plausible explanation for our findings, suggesting that a moderate dose of alcohol use may mitigate feelings of psychological distress [74]. Still, at higher doses, alcohol may provide less protection from irritability once inhibitory control declines, often giving way to overt aggression particularly in men with heightened irritability [75]. An alternative explanation may be that the findings are confounded by other sample characteristics [76], for instance more well-adjusted respondents may cluster in the ‘moderate group’. This latter explanation seems less probable, given that moderate drinkers seem less resilient in their efforts to abstain from alcohol use (as shown in Fig. 1, panel a).

### Strengths and limitations

An important limitation is the relatively small sample size by comparison to epidemiological studies in this area. Our sample size was adequately powered to undertake factor analysis based on Bryant and Yarnold’s criteria [77], which would require 5 respondents per item (*N* = 130). Still, we note that there are divergent views about sample size calculations for factor analysis, and others suggest a minimum of 500 participants [78]. Other considerations to note about the generalizability of these findings concern the outpatient setting, with a majority of respondents whose primary reason for treatment related to opiate use. Although nearly half of all respondents reported using alcohol, and some at a very severe level, we noted that alcohol use was weakly correlated with only 2 SDS items, and did not load onto factor 2. It may be that including more participants from alcohol detox or inpatient settings could render different patterns of correlation. It is also possible that the method of administration of the SDS measure may have influenced the strength of correlations. SDS was rated for the primary substance of concern, which in some cases could have been a substance other than alcohol (e.g., heroin), and this may have therefore impacted on the strength of observed correlations between alcohol use and SDS. Finally, we also note that the cross-sectional nature of these data limit the possibility of making more certain claims about casual relationships. Our deductions from this sample should therefore be taken as a preliminary investigation of functional links between aspects of psychopathology and addictive behaviours, awaiting further validation in prospective studies with mediational tests.

### Considerations for practice and research

The emerging literature on comorbidity suggests that substance induced depression and anxiety symptoms are relatively uncommon, though they may be more conspicuous in addiction treatment settings. Our findings show that after factoring out general psychological distress, essentially no specific covariance between substance use patterns and psychological distress items remains. This is strong evidence that general psychological distress is a moderator of the relationship, but it is unclear from the current study in which direction the causal arrow points.

From this perspective, the common practice of assuming that most CMD symptoms are drug-induced is ethically questionable, especially if such practices hamper timely diagnosis and access to mental healthcare. Psychometrically adequate screening methods are available to detect CMD in substance users [27, 41, 79, 80], but these methods are not consistently implemented in routine practice. Based on our symptom-level correlations, we propose that diagnostic results may perhaps be enhanced by applying a repeated screening method after a month of watchful wait for heavy cannabis users who show signs of severe disruption to concentration, and heavy drinkers who show increased signs of irritability and hyper-arousal. Residual anxiety symptoms that may reflect craving/withdrawal phenomena should be carefully distinguished from generalised anxiety disorder, possibly by supplementing screening measures with probing questions or interviews. The training of addiction treatment professionals [81, 82] in the application of such screening methods may be an important focus of future dissemination studies and policies.

## Conclusion

After controlling for latent levels of psychopathology, we found little evidence of associations between symptoms of depression/anxiety with substance use. The only significant symptom-level associations were impaired concentration linked to cannabis use and irritability linked to alcohol use. Severity of dependence –a psychological construct– is prominently associated with depressive rumination, and negatively correlated with residual anxiety symptoms related to substance use (e.g., craving). Overall, this analysis supports a psychological understanding of comorbidity; with dependence, craving, negative reinforcement and rumination as key variables.

## Abbreviations

CMD, common mental disorders; GAD-7, generalized anxiety disorder questionnaire (7-item anxiety scale); PHQ-9, patient health questionnaire (9-item depression scale); PROMs, patient reported outcome measures; QF, quantity x frequency (of substance use); SDS, severity of dependence scale; TOP, Treatment Outcomes Profile questionnaire
